# A Combined Association Mapping and Linkage Analysis of Kernel Number Per Spike in Common Wheat (*Triticum aestivum* L.)

**DOI:** 10.3389/fpls.2017.01412

**Published:** 2017-08-18

**Authors:** Weiping Shi, Chenyang Hao, Yong Zhang, Jingye Cheng, Zheng Zhang, Jian Liu, Xin Yi, Xiaoming Cheng, Daizhen Sun, Yanhao Xu, Xueyong Zhang, Shunhe Cheng, Pingyi Guo, Jie Guo

**Affiliations:** ^1^College of Agronomy, Shanxi Agricultural University Jinzhong, China; ^2^Key Laboratory of Crop Gene Resources and Germplasm Enhancement, Ministry of Agriculture, The National Key Facility for Crop Gene Resources and Genetic Improvement, Institute of Crop Science, Chinese Academy of Agricultural Sciences Beijing, China; ^3^Key Laboratory of Wheat Biology and Genetic Improvement for Low and Middle Yangtze Valley (Ministry of Agriculture), Lixiahe Agricultural Institute of Jiangsu Province Yangzhou, China; ^4^College of Agronomy, Yangzhou University Yangzhou, China; ^5^Hubei Collaborative Innovation Centre for Grain Industry and College of Agriculture, Yangtze University Jingzhou, China

**Keywords:** bi-parental population analysis, GWAS, iSelect wheat 90K SNP chip

## Abstract

Kernel number per spike (KNPS) in wheat is a key factor that limits yield improvement. In this study, we genotyped a set of 264 cultivars, and a RIL population derived from the cross Yangmai 13/C615 using the 90 K wheat iSelect SNP array. We detected 62 significantly associated signals for KNPS at 47 single nucleotide polymorphism (SNP) loci through genome-wide association analysis of data obtained from multiple environments. These loci were on 19 chromosomes, and the phenotypic variation attributable to each one ranged from 1.53 to 39.52%. Twelve (25.53%) of the loci were also significantly associated with KNPS in the RIL population grown in multiple environments. For example, *BS00022896_51-2A*_*TT*_, *BobWhite_c10539_201-2D*_*AA*_, *Excalibur_c73633_120-3B*_*GG*_, and *Kukri_c35508_426-7D*_*TT*_ were significantly associated with KNPS in all environments. Our findings demonstrate the effective integration of association mapping and linkage analysis for KNPS, and underpin KNPS as a target trait for marker-assisted selection and genetic fine mapping.

## Introduction

Yield improvement is an on-going endeavor in wheat breeding. Wheat yield is determined by spike number per unit area, kernel number per spike (KNPS) and thousand kernel weight. Increased yield mainly depends on increased KNPS when the other two parameters are unchanged (Slafer and Andrade, 1989; Fischer, 2008, 2011; Dobrovolskaya et al., 2015). Thus, molecular interpretation of the inheritance mechanism of KNPS is of significance for marker-assisted selection and molecularly designed wheat breeding.

The major methods for genetic dissection of complex traits in crop species include family-based linkage mapping and association mapping of germplasm collections (Mackay and Powell, 2007; Cadic et al., 2013). Association mapping has three advantages compared to conventional linkage mapping: (1) it saves time and cost of construction of suitable segregating populations, and by using existing populations there can be a wide diversity of materials; (2) it is able to detect multi-allelic variation, and thus helps to identify the most favorable alleles contributing to a target trait in a single analysis; and (3) its higher resolution is more powerful for fine mapping of quantitative trait loci (QTLs; Breseghello and Sorrells, 2006; Atwell et al., 2010). Research across many crops has shown that association analysis is a promising method for mining favorable alleles, despite some limitations. For example, association analysis is less efficient than linkage analysis for study of species with low genetic diversity (Zhao et al., 2007; Myles et al., 2009). However, association analysis and linkage mapping are complementary methods, and their combination can be used for cross-validation (Nordborg and Weigel, 2008). Thus, an integrated application of both methods is more efficient in discovering and validating QTLs in crop species (Zhang et al., 2011).

Since Paterson et al. (1988) first used restriction fragment length polymorphism (RFLP) to map QTLs for fruit mass, soluble solids concentration, and pH in tomatoes, more than 100 QTLs for KNPS distributed on all 21 chromosomes in wheat have been reported (Börner et al., 2002; Huang et al., 2004, 2006; Narasimhamoorthy et al., 2006; Kirigwi et al., 2007; Deng et al., 2011; Cui et al., 2012, 2014; Wu et al., 2012; Jia et al., 2013; Zhang et al., 2016). Börner et al. (2002) and Kirigwi et al. (2007) used recombinant inbred line (RIL) populations and both studies detected KNPS-related QTLs on chromosomes 4A and 7D. Advanced backcross populations were used in detecting KNPS-related QTLs in chromosomes 1D, 2A, 3B, 3D, 6A, 7A, and 7D (Huang et al., 2004; Narasimhamoorthy et al., 2006). QTLs for KNPS were identified on chromosomes 1A, 2A, 2B, 2D, 3A, 4A, 4D, 6A, 6B, and 7B using doubled haploid populations (McCartney et al., 2005; Huang et al., 2006; Heidari et al., 2011; Zhang et al., 2016). Using an F_2:3_ population of 237 families Wang et al. (2011) located QTLs on chromosomes 1A, 2D, 3B, 4A, 4B, 5A, 5B, 7A, and 7B. Through genome-wide association analysis based on a Chinese wheat mini core collection Zhang et al. (2012) detected 23 KNPS-associated loci on chromosomes 1A, 1B, 1D, 2A, 2B, 3A, 3B, 3D, 4D, 5B, 5D, 6A, 6B, and 6D. Guo et al. (2015a,b) identified 13 KNPS-associated loci on chromosomes 1A, 1B, 1D, 2D, 3B, 5B, 5D, 6B, and 7B using a set of wheat cultivars. All KNPS-associated QTLs reported so far in wheat have been mapped and located using RFLP and simple sequence repeat (SSR) markers. Although these findings provide valuable information, the marker densities proved insufficient for both marker assisted selection and gene isolation (Devos et al., 1993; Röder et al., 1998; Somers et al., 2004; Torada et al., 2006).

With recent developments in wheat gene chip technology and reduced of sequencing costs, single nucleotide polymorphism (SNP) markers have been extensively adopted due to their high density, representativeness, stable inheritance, and capability of automatic detection (Allen et al., 2011; Cavanagh et al., 2013). In particular, the 90K SNP GoldenGate chip based on the Illumina platform has been widely applied in detection of polymorphisms in both tetraploid and hexaploid wheat (Akhunov et al., 2009; Lai et al., 2012). The iSelect wheat 90K SNP chip has been used to discover yield-related QTLs in wheat. For example, an F_8_-generation RIL population derived from the cross Zhou 8425B/Chinese Spring was used by Gao et al. (2015) to identify 24 yield-related QTLs, of which five loci (*QGC-W.caas-7AL, QNDVIS.caas-7AL, QGC-S.caas-3AS, QCTD-A.caas-5BS*, and *QCTD-10.caas-5BS*) were detected simultaneously in multiple environments. Through genome-wide association analyses, Zanke et al. (2015) detected 58 loci significantly associated with thousand kernel weight and distributed in all chromosomes except 4D and 5D, and Ain et al. (2015) detected 44 loci significantly associated with yield (grain number per spike, thousand grain weight, grain yield, biological yield, and harvest index) in germplasm sets. As these genome-wide association studies were based on genetic resource collections and focused on mining of yield-related genes or loci the individual findings still need validation by linkage analysis in segregating populations.

In this study, a set of 264 wheat cultivars, and a RIL population with 198 lines derived from the cross Yangmai 13/C615 were genotyped using iSelect wheat 90K SNP high-density chips. Together with the KNPS phenotypic data detected in multiple environments, we confirmed the presence of KNPS-related loci and identified favorable alleles through an integration of association mapping and linkage analysis. The findings provide useful information for marker-assisted selection of KNPS in wheat.

## Materials and methods

### Plant materials

The material for association analysis consisted of 264 wheat cultivars, including 258 from China and six from other countries. The six introduced accessions included three from Italy, one from Mexico, one from Chile, and one from Japan. The 258 domestic accessions (Table S1) were collected from Jiangsu (65 accessions), Henan (36), Shandong (22), Shaanxi (29), Sichuan (18), Anhui (16), Beijing (12), Hunan (12), Hebei (9), Hubei (7), Gansu (5), Zhejiang (4), Fujian (4), Shanxi (4), Guizhou (3), Heilongjiang (2), Jiangxi (1), and Yunnan (1) or were of unknown origin (8).

Yangmai 13, bred by Lixiahe Agricultural Institute in Jiangsu province, has been extensively promoted in the Lower and Middle Yangtze River Valley winter wheat region and C615 is a synthetic wheat accession [durum cultivar CEAT × AE. SQUARROSA (895)] introduced from the International Maize and Wheat Improvement Center (CIMMYT), Mexico. The average KNPS for Yangmai 13 exceeds that of C615 (57.9 vs. 47.0; Table 1). The F_7_ RIL population of 198 lines was developed by single seed descent from an initial F_2_ population.

**Table 1:** Descriptive statistics for kernel number per spike in the two populations assessed in this study.

**Population type**		**Environment**	**Mean**	**SD^a^**	**Min**	**Max**	**CV^b^ (%)**
Germplasm set		14JZ	54.95	7.60	33.17	94.23	13.83
		14YZ	57.76	7.63	36.60	95.40	13.21
		15JZ	51.19	5.42	36.95	68.75	10.59
		15YZ	54.31	6.48	41.40	79.60	11.93
		16XX	58.91	8.74	35.80	85.80	14.84
		BLUP	55.41	5.31	41.05	83.17	9.58
RIL population		15YZ	57.32	5.63	43.20	74.80	9.82
		16JZ	43.42	4.75	29.43	57.14	10.94
		16YZ	57.80	5.60	44.67	72.67	9.69
		BLUP	52.94	4.01	40.73	65.98	7.58
RIL parents	C615	15YZ	48.00	–	–	–	–
		16JZ	42.00	–	–	–	–
		16YZ	51.00	–	–	–	–
		Mean	47.00	–	–	–	–
	Yangmai 13	15YZ	60.25	–	–	–	–
		16JZ	51.14	–	–	–	–
		16YZ	62.40	–	–	–	–
		Mean	57.93	–	–	–	–

a *SD, standard deviation*.

b *CV, coefficient of variation*.

### Phenotyping

The cultivar set was planted in 2013–2014 and 2014–2015 at Jingzhou in Hubei province and Yangzhou in Jiangsu, and in 2015–2016 at Xinxiang in Henan; these environments were designated 14JZ, 14YZ, 15JZ, 15YZ, and 16XX, respectively. The field trials were grown as thrice-replicated randomized blocks. Lines in each replicate were planted in 3-row plots at a density of 40 kernels/133 cm row, and a row spacing of 25 cm. Plant densities were thinned to around 30 at the seedling stage. Thirty spikes of each line were randomly selected from the middle row and used to score KNPS. Field management followed local practices.

The Yangmai 13/C615 RIL population was planted in 2014–2015 at Yangzhou, and in 2015–2016 at Yangzhou and Jingzhou. These environments were named 15YZ, 16JZ, and 16YZ, respectively. The field experiments were designed as randomized blocks with three replicates. Plot sizes, plant densities, and spike sampling were similar to those described for the cultivars.

### Genotyping and data analysis

Genomic DNA was extracted from the test materials using the CTAB method (Sharp et al., 1989). Statistical analyses were conducted on SPSS 21.0 (http://www.brothersoft.com/ibm-spss-statistics-469577.html). The mean KNPS was computed by a best linear unbiased predictor (BLUP) method (Bernardo, 1996a,b; Bernardo et al., 1996).

SNP markers were detected by the Biotechnology Center, Department of Plants, University of California, USA, by using the Illumina SNP genotyping platform and BeadArray Microbead Chip (Cavanagh et al., 2013). SNP allele clustering and genotype calling were conducted on Genomestudio v2011.1 (Wang et al., 2014). Chromosome position of SNP markers are provided in Cavanagh et al. (2013).

Genetic diversity of SNP markers was analyzed on PowerMarker 3.25 (Liu and Muse, 2005). Genetic structure of the cultivar set was evaluated by Structure 2.3.2 using 3,656 SNP markers distributed on all 21 wheat chromosomes (Pritchard et al., 2000). The number of subpopulations was determined by a ΔK model (Evanno et al., 2005). Genome-wide association analysis of KNPS with SNP markers was based on a Q+K model (Yu et al., 2005; Zhang et al., 2010) and TASSEL 5.0 (Bradbury et al., 2007; http://www.maizegenetics.net/). SNP loci at frequencies lower than 0.05 were not considered, the threshold *P* of association signals was set as the 1/SNP marker number (1/20,037 = 4.99 × 10^−5^), or namely *P* < 4.99 × 10^−5^, −Log *P* > 4.30. The genetic effects of favorable alleles at associated loci in the cultivar set, and in the RIL population were tested via ANOVA on SPSS 21.0.

## Results

### Phenotypic assessment of the cultivar population and rils

Analysis of KNPS in the cultivar population grown in five environments (14JZ, 14YZ, 15JZ, 15YZ, 16XX) and best linear unbiased predictions (BLUP) indicated coefficients of variation of KNPS data in the range 9.58–14.84%. Between-environment correlation coefficients for KNPS varied between 0.521 and 0.874 (*P* < 0.001) for cultivars, compared to 0.513 and 0.833 (*P* < 0.001) for the RIL population (Table S2). Although the coefficient of variation of KNPS for the RIL population of 7.58–10.94% was less than the cultivar population the variation was still quite rich (Table 1).

### Allelic diversity and genetic structure analysis

The genetic diversity of the cultivar population was analyzed using 22,325 SNP markers. The major allele frequency (MAF) varied from 0.500 to 0.998 (mean 0.785), polymorphism information content (PIC) varied between 0.004 and 0.375 (mean 0.238), and gene diversity varied between 0.004 and 0.500 (mean 0.294; Table S3), indicating this set of cultivars has high genetic diversity at the SNP level.

**Figure 1:**
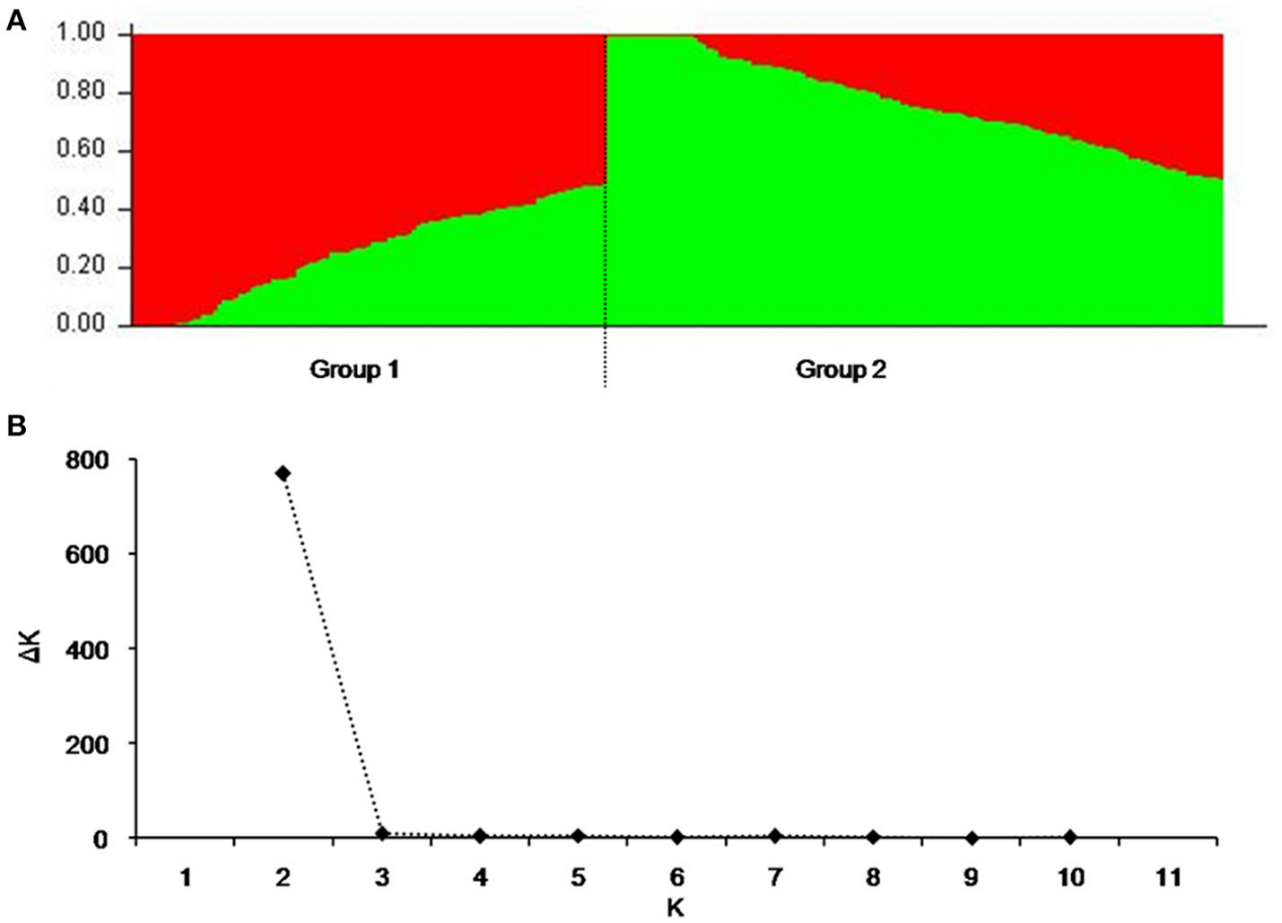
Population structure of 264 released cultivars based on 3,656 SNP markers with a whole-genome coverage. **(A)** Genetic structure produced by Structure V2.3.2, **(B)** Number of sub-populations estimated by ΔK at a range of K-values.

To reduce false associations, genetic structure (Q-value) and between-individual relationship coefficient (K-value) of the cultivar population were determined. The population divided into two subpopulations (Figure 1A), and ΔK was maxim at K = 2, further validating the above subdivision (Figure 1B).

### Genome-wide association studies on KNPS with SNP markers in the cultivar population

**Figure 2:**
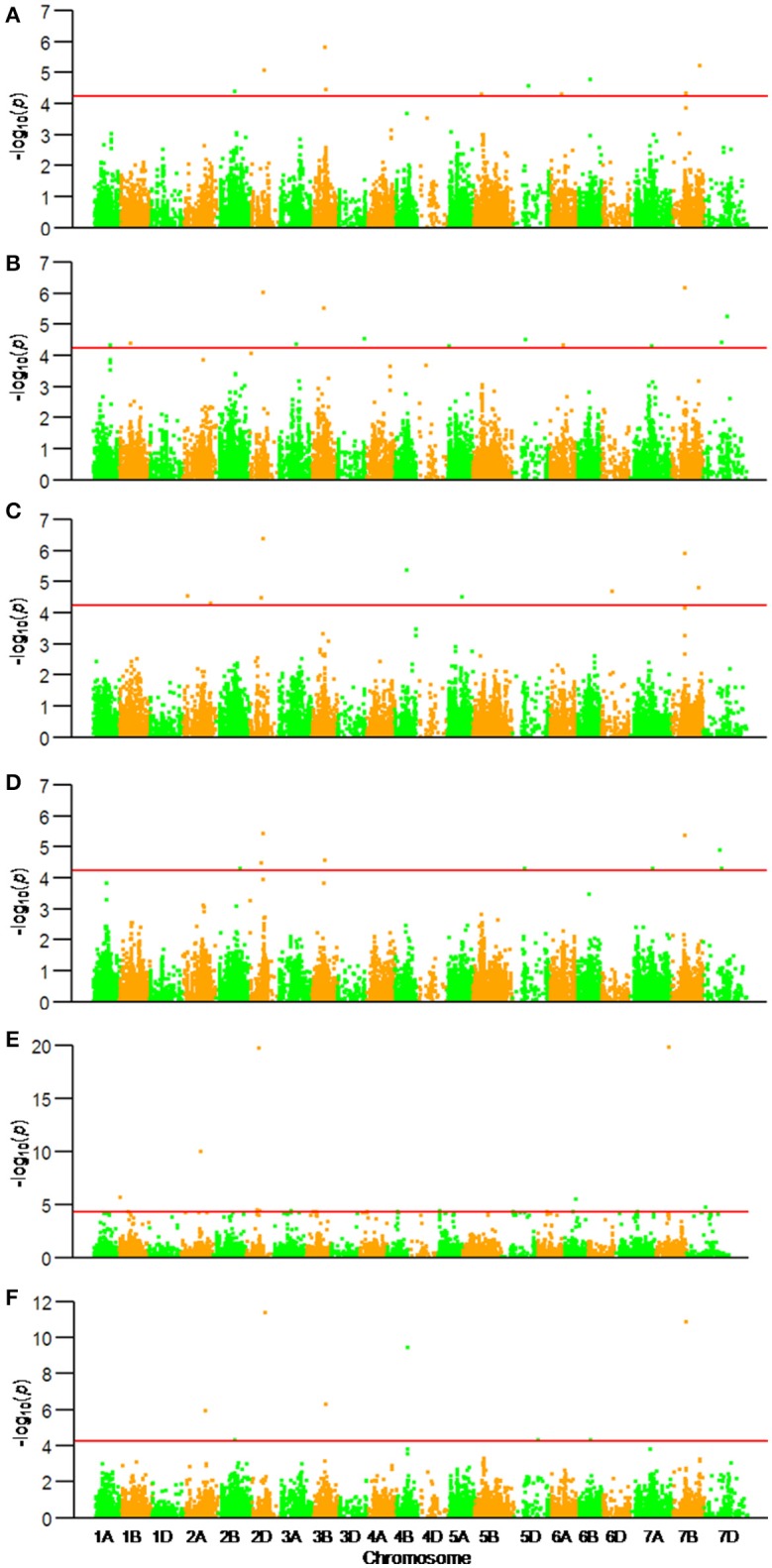
Associations of kernel number per spike with 20,037 genome-wide SNP markers illustrated as dotplots of compressed MLM at *P* < 4.99 × 10^−5^. Red dotted line indicates the threshold value for declaring a significant association. **(A)** 14JZ; **(B)** 14YZ; **(C)** 15JZ; **(D)** 15YZ; **(E)** 16XX; **(F)** BLUP.

Among the 22,325 SNP markers 20,037 had frequencies above 0.05. Association analysis between KNPS and SNPs detected 62 significantly-associated signals at 47 loci (*P* < 4.99 × 10^−5^). The associated loci were distributed across all chromosomes except 1D and 4D, and the explained phenotypic variation (*R*^2^) ranging from 1.53–39.52% (Figure 2, Table 2). SNPs *BS00022896_51* (2A), *BobWhite_c10539_201* (2D), *Excalibur_c73633_120* (3B), *BS00063906_51* (6B), and *GENE-4456_153* (7B) were significantly associated with KNPS in two or more environments (Table 2).

**Table 2:** Sixty-two significant association signals (P<4.99×10^−5^) involving 47 SNP markers.

**SNP name**	**Chr**.	**Position**	**Alleles**	**Environment**	***P-*value**	***R*^2^**
*wsnp_Ex_c41953_48657850*	1A	106.27	T/C	14YZ	4.84 × 10^−5^	6.77
*BS00010868_51*	1B	9.68	T/C	16XX	2.24 × 10^−6^	12.49
*Kukri_rep_c101799_95*	1B	64.46	A/C	14YZ	4.21 × 10^−5^	3.78
*GENE-1086_1111*	2A	25.97	A/C	15JZ	2.87 × 10^−5^	1.84
*BS00022896_51*	2A	109.52	T/C	16XX	9.69 × 10^−11^	18.75
				BLUP	1.20 × 10^−6^	13.30
*Tdurum_contig92425_1612*	2A	151.57	A/G	15JZ	4.98 × 10^−5^	12.04
*BS00014345_51*	2B	88.86	T/C	BLUP	4.81 × 10^−5^	3.64
*BS00037278_51*	2B	96.14	A/G	14JZ	4.22 × 10^−5^	2.09
*wsnp_JD_c12346_12606967*	2B	130.62	A/G	15YZ	4.98 × 10^−5^	8.04
*D_contig13956_175*	2D	63.47	A/G	16XX	3.53 × 10^−5^	9.43
*D_contig35123_619*	2D	66.03	A/G	15YZ	3.36 × 10^−5^	8.34
*D_F5XZDLF02JSEI4_223*	2D	66.03	A/G	15JZ	3.43 × 10^−5^	3.83
*BobWhite_c10539_201*	2D	77.80	A/G	14JZ	8.55 × 10^−6^	12.79
				14YZ	9.64 × 10^−7^	13.31
				15JZ	4.34 × 10^−7^	15.57
				15YZ	3.76 × 10^−6^	1.53
				16XX	1.99 × 10^−20^	39.52
				BLUP	4.32 × 10^−12^	23.71
*wsnp_Ex_c12223_19533198*	2D	82.82	A/G	16XX	4.51 × 10^−5^	6.37
*wsnp_JD_c43971_30568640*	3A	109.95	T/C	14YZ	4.55 × 10^−5^	4.51
*Ra_c3994_598*	3A	109.95	T/C	16XX	4.35 × 10^−5^	8.42
*Excalibur_c73633_120*	3B	65.55	A/G	14JZ	1.59 × 10^−6^	15.52
				14YZ	3.14 × 10^−6^	13.93
				BLUP	5.22 × 10^−7^	13.58
*BS00066466_51*	3B	71.34	T/C	15YZ	2.68 × 10^−5^	2.26
*Kukri_c35105_294*	3B	73.94	T/G	14JZ	3.53 × 10^−5^	3.34
*Kukri_c18420_705*	3D	148.48	T/C	14YZ	3.03 × 10^−5^	3.74
*tplb0051b16_1324*	4A	48.98	A/G	16XX	4.89 × 10^−5^	7.35
*BS00075746_51*	4B	61.84	T/C	BLUP	3.57 × 10^−10^	6.68
*Tdurum_contig54854_547*	4B	65.59	A/G	15JZ	4.30 × 10^−6^	8.38
*Excalibur_c29933_351*	5A	13.42	T/C	16XX	4.48 × 10^−5^	7.56
*RAC875_c84991_116*	5A	15.58	A/C	14YZ	4.98 × 10^−5^	7.35
*BS00064412_51*	5A	15.61	T/C	16XX	3.99 × 10^−5^	4.16
*Tdurum_contig10759_260*	5A	84.13	A/G	15JZ	3.16 × 10^−5^	8.73
*Tdurum_contig13025_774*	5B	40.57	A/C	14JZ	4.98 × 10^−5^	7.35
*D_contig24916_701*	5D	70.11	A/G	15YZ	4.98 × 10^−5^	7.35
*D_contig65543_191*	5D	73.99	A/C	14YZ	3.22 × 10^−5^	8.93
*Kukri_rep_c102792_163*	5D	83.51	T/C	14JZ	2.79 × 10^−5^	8.16
*BobWhite_c3750_335*	5D	136.83	A/C	BLUP	4.98 × 10^−5^	7.35
*Tdurum_contig11413_700*	6A	63.70	A/G	14JZ	4.98 × 10^−5^	7.35
*Ra_c12362_422*	6A	79.08	A/C	14YZ	4.81 × 10^−5^	7.51
*Tdurum_contig63539_178*	6A	82.38	T/C	16XX	4.98 × 10^−5^	7.35
*BS00063906_51*	6B	59.92	T/G	14JZ	1.76 × 10^−5^	1.82
				16XX	3.24 × 10^−6^	16.70
				BLUP	4.69 × 10^−5^	16.47
*Tdurum_contig29027_92*	6D	68.00	A/G	15JZ	2.07 × 10^−5^	8.11
*D_GDEEGVY01CQJ66_272*	7A	127.75	T/C	14YZ	4.98 × 10^−5^	7.35
*wsnp_BF474379A_Ta_2_2*	7A	135.81	T/C	15YZ	4.98 × 10^−5^	7.35
*GENE-4456_153*	7B	72.74	T/C	14JZ	4.81 × 10^−5^	3.54
				14YZ	7.08 × 10^−7^	15.19
				15JZ	1.29 × 10^−6^	2.81
				15YZ	4.21 × 10^−6^	13.55
				16XX	1.45 × 10^−20^	29.09
				BLUP	1.37 × 10^−11^	26.72
*IACX5767*	7B	150.60	A/G	15JZ	1.58 × 10^−5^	7.39
*BS00003630_51*	7B	150.60	T/C	14JZ	6.27 × 10^−6^	7.62
*D_GDS7LZN02H6ID8_55*	7D	91.57	T/G	15YZ	1.27 × 10^−5^	7.52
*CAP7_c1383_548*	7D	101.06	A/G	14YZ	3.79 × 10^−5^	2.98
*Kukri_c35508_426*	7D	102.12	T/C	15YZ	4.98 × 10^−5^	7.35
*D_GA8KES401AVKPJ_56*	7D	106.28	A/C	16XX	2.08 × 10^−5^	3.43
*D_contig10938_340*	7D	135.55	T/C	14YZ	5.83 × 10^−6^	9.12

### Favorable alleles and their genetic effects

The genetic effects of alleles at the 47 associated loci (Table 3) ranged from 0.45 to 3.68, indicating positive effects on KNPS. *GENE-4456_153-7B*_*TT*_ has the largest effect (2.80 kernels per spikelet, 14JZ; 2.24 kernels, 14YZ; 3.25 kernels, 15JZ; 2.51 kernels, 15YZ; 3.68 kernels, 16XX) and was detected in all environments (Table 3). Moreover, the frequency of favorable alleles at associated loci varied from 8.33 to 92.82%. The frequencies of 19 favorable alleles exceeded 50% and were distributed in obviously skewed ways, indicative of prior strong selection in breeding programs.

**Table 3:** Favorable alleles and effects of 47 SNP loci significantly (*P* < 4.99 × 10^−5^) associated with kernel number per spike in the germplasm set.

**SNP name**	**Chr**.	**Position (cM)**	**Favorable allele**	**Freq. (%)**	**Allele effect**					
					**14JZ**	**14YZ**	**15JZ**	**15YZ**	**16XX**	**Average**
*wsnp_Ex_c41953_48657850*	1A	106.27	TT	19.70		0.61				
*BS00010868_51*	1B	9.68	CC	34.85					1.42	
*Kukri_rep_c101799_95*	1B	64.46	AA	37.44		1.54				
*GENE-1086_1111*	2A	25.97	AA	8.33			1.11			
*BS00022896_51*	2A	109.52	TT	64.82					2.78	1.16
*Tdurum_contig92425_1612*	2A	151.57	AA	36.89			1.45			
*BS00014345_51*	2B	88.86	CC	65.53						1.19
*BS00037278_51*	2B	96.14	AA	50.00	1.10					
*wsnp_JD_c12346_12606967*	2B	130.62	AA	36.89				0.45		
*D_contig13956_175*	2D	63.47	GG	35.96					0.52	
*D_contig35123_619*	2D	66.03	GG	67.42				1.14		
*D_F5XZDLF02JSEI4_223*	2D	66.03	GG	66.29			1.15			
*BobWhite_c10539_201*	2D	77.80	AA	70.45	1.39	1.50	1.15	1.19	2.92	1.20
*wsnp_Ex_c12223_19533198*	2D	82.82	GG	46.32					0.86	
*wsnp_JD_c43971_30568640*	3A	109.95	CC	66.67		1.33				
*Ra_c3994_598*	3A	109.95	TT	37.00					1.54	
*Excalibur_c73633_120*	3B	65.55	GG	92.82	2.04	2.11				2.02
*BS00066466_51*	3B	71.34	TT	36.96				1.65		
*Kukri_c35105_294*	3B	73.94	TT	65.91	1.40					
*Kukri_c18420_705*	3D	148.48	TT	67.42		1.13				
*tplb0051b16_1324*	4A	48.98	GG	36.59					1.47	
*BS00075746_51*	4B	61.84	CC	69.55						2.90
*Tdurum_contig54854_547*	4B	65.59	GG	66.67			1.10			
*Excalibur_c29933_351*	5A	13.42	TT	36.32					1.54	
*RAC875_c84991_116*	5A	15.58	CC	36.89		1.45				
*BS00064412_51*	5A	15.61	CC	67.42					1.20	
*Tdurum_contig10759_260*	5A	84.13	AA	36.95			0.61			
*Tdurum_contig13025_774*	5B	40.57	AA	36.89	0.45					
*D_contig24916_701*	5D	70.11	GG	36.89				1.45		
*D_contig65543_191*	5D	73.99	AA	35.32		0.60				
*Kukri_rep_c102792_163*	5D	83.51	CC	66.67	1.26					
*BobWhite_c3750_335*	5D	136.83	AA	36.89						0.45
*Tdurum_contig11413_700*	6A	63.70	AA	36.89	1.45					
*Ra_c12362_422*	6A	79.08	AA	36.76		0.51				
*Tdurum_contig63539_178*	6A	82.38	CC	36.89					1.40	
*BS00063906_51*	6B	59.92	GG	50.00	1.88				1.96	1.72
*Tdurum_contig29027_92*	6D	68.00	GG	67.05			1.15			
*D_GDEEGVY01CQJ66_272*	7A	127.75	CC	36.89		1.45				
*wsnp_BF474379A_Ta_2_2*	7A	135.81	CC	36.89				1.45		
*GENE-4456_153*	7B	72.74	TT	41.29	2.80	2.24	3.25	2.51	3.68	2.67
*IACX5767*	7B	150.60	GG	11.41			1.50			
*BS00003630_51*	7B	150.60	TT	61.45	1.50					
*D_GDS7LZN02H6ID8_55*	7D	91.57	GG	67.80				1.20		
*CAP7_c1383_548*	7D	101.06	AA	61.36		1.11				
*Kukri_c35508_426*	7D	102.12	TT	36.89				1.45		
*D_GA8KES401AVKPJ_56*	7D	106.28	CC	66.29					1.12	
*D_contig10938_340*	7D	135.55	CC	64.77		1.19				

### Overlapping between association signals and linkage analysis

To validate the effectiveness of associated loci in the germplasm set, we used the same iSelect wheat 90K SNP chip to scan the RIL population. Among the 47 associated loci found earlier 16 (34.04%) were polymorphic between Yangmai 13 and C615. Furthermore, the genetic effects of the favorable alleles in the cultivar population were analyzed by ANOVA in the RIL population. Twelve loci (25.53% of all associated loci) were significantly correlated with KNPS in multiple environments (*P* < 0.05). In particular, four favorable alleles, *BS00022896_51-2A*_*TT*_, *BobWhite_c10539_201-2D*_*AA*_, *Excalibur_c73633_120-3B*_*GG*_, and *Kukri_c35508_426-7D*_*TT*_ were significantly associated with KNPS in all environments. *BS00022896_51-2A*_*TT*_ had a large genetic effect on KNPS (2.09 kernels, 15YZ; 1.05 kernels, 16JZ; 1.28 kernels, 16YZ; 1.22 kernels, BLUP; Table 4). Moreover, analysis of the 12 SNP loci showed that the favorable alleles at those loci were identical in both populations, indicating that these alleles had consistent effects in both populations.

**Table 4:** Favorable alleles and effects of 12 SNP loci validated in the RIL population.

**SNP name**	**Chr**.	**Environment**	**Allele**	**Mean ± SE**	**Allele effect**	***P-*value**
*GENE-1086_1111*	2A	15YZ	AA	58.67 ± 0.57	1.26	2.00 × 10^−3^
			Others	56.34 ± 0.48		
		16YZ	AA	59.16 ± 0.58	1.29	1.41 × 10^−3^
			Others	56.76 ± 0.47		
		Average	AA	53.78 ± 0.39	0.84	3.29 × 10^−3^
			Others	52.22 ± 0.34		
*BS00022896_51*	2A	15YZ	TT	59.30 ± 0.69	2.09	3.56 × 10^−4^
			Others	56.37 ± 0.43		
		16JZ	TT	44.55 ± 0.61	1.05	4.30 × 10^−2^
			Others	43.09 ± 0.38		
		16YZ	TT	59.05 ± 0.64	1.28	3.10 × 10^−2^
			Others	57.25 ± 0.46		
		Average	TT	54.09 ± 0.49	1.22	5.00 × 10^−3^
			Others	52.38 ± 0.32		
*BS00014345_51*	2B	16JZ	CC	44.05 ± 0.37	0.59	3.70 × 10^−2^
			Others	42.69 ± 0.56		
		16YZ	CC	58.61 ± 0.47	0.81	1.20 × 10^−3^
			Others	56.71 ± 0.60		
		Average	CC	53.36 ± 0.31	0.49	3.40 × 10^−2^
			Others	52.20 ± 0.47		
*wsnp_JD_c12346_12606967*	2B	15YZ	AA	57.76 ± 0.43	0.45	4.00 × 10^−3^
			Others	54.44 ± 0.96		
		16YZ	AA	58.10 ± 0.41	0.32	4.20 × 10^−2^
			Others	55.74 ± 1.26		
		Average	AA	53.25 ± 0.31	0.34	3.00 × 10^−3^
			Others	50.77 ± 0.64		
*BobWhite_c10539_201*	2D	15YZ	AA	57.71 ± 0.43	0.43	8.00 × 10^−3^
			Others	54.70 ± 0.96		
		16JZ	AA	43.96 ± 0.35	0.36	3.00 × 10^−3^
			Others	41.12 ± 0.65		
		16YZ	AA	58.44 ± 0.39	0.57	4.80 × 10^−5^
			Others	53.79 ± 1.00		
		Average	AA	53.23 ± 0.31	0.31	8.00 × 10^−3^
			Others	51.08 ± 0.69		
*wsnp_Ex_c12223_19533198*	2D	15YZ	GG	58.10 ± 0.41	0.46	4.00 × 10^−3^
			Others	55.04 ± 0.99		
		16YZ	GG	43.96 ± 0.35	0.33	3.90 × 10^−2^
			Others	42.42 ± 0.78		
		Average	GG	53.56 ± 0.28	0.35	1.00 × 10^−3^
			Others	51.23 ± 0.68		
*Excalibur_c73633_120*	3B	15YZ	GG	57.83 ± 0.43	0.46	6.00 × 10^−3^
			others	54.83 ± 0.89		
		16JZ	GG	43.93 ± 0.36	0.46	1.00 × 10^−3^
			others	40.76 ± 0.67		
		16YZ	GG	58.51 ± 0.40	0.59	7.87 × 10^−5^
			others	54.16 ± 0.90		
		Average	GG	53.33 ± 0.31	0.38	1.00 × 10^−3^
			others	50.83 ± 0.62		
*tplb0051b16_1324*	4A	15YZ	GG	58.52 ± 0.52	1.17	1.00 × 10^−3^
			others	56.10 ± 0.54		
		16JZ	GG	44.50 ± 0.40	1.06	1.00 × 10^−3^
			others	42.35 ± 0.47		
		Average	GG	53.77 ± 0.38	0.80	2.00 × 10^−3^
			others	52.12 ± 0.37		
*RAC875_c84991_116*	5A	15YZ	CC	58.59 ± 0.62	1.30	3.00 × 10^−3^
			others	56.30 ± 0.48		
		16YZ	CC	59.36 ± 0.62	1.52	1.00 × 10^−3^
			others	56.69 ± 0.46		
		Average	CC	53.87 ± 0.42	0.95	2.00 × 10^−3^
			others	52.20 ± 0.35		
*wsnp_BF474379A_Ta_2_2*	7A	15YZ	CC	58.68 ± 0.55	1.32	3.48 × 10^−4^
			others	56.06 ± 0.46		
		16YZ	CC	58.85 ± 0.51	0.98	9.00 × 10^−3^
			others	56.93 ± 0.52		
		Average	CC	53.68 ± 0.38	0.72	8.00 × 10^−3^
			others	52.28 ± 0.36		
*BS00003630_51*	7B	15YZ	TT	57.94 ± 0.43	0.41	7.00 × 10^−3^
			others	54.87 ± 0.77		
		16JZ	TT	43.94 ± 0.34	0.36	5.00 × 10^−3^
			others	41.29 ± 0.78		
		Average	TT	53.44 ± 0.29	0.32	2.00 × 10^−3^
			others	51.05 ± 0.55		
*Kukri_c35508_426*	7D	15YZ	TT	59.43 ± 0.57	1.97	1.24 × 10^−6^
			others	55.77 ± 0.46		
		16JZ	TT	44.38 ± 0.39	0.82	1.90 × 10^−2^
			others	42.85 ± 0.49		
		16YZ	TT	58.99 ± 0.54	1.23	3.00 × 10^−3^
			others	56.69 ± 0.52		
		Average	TT	54.10 ± 0.35	1.16	6.16 × 10^−5^
						

**Figure 3:**
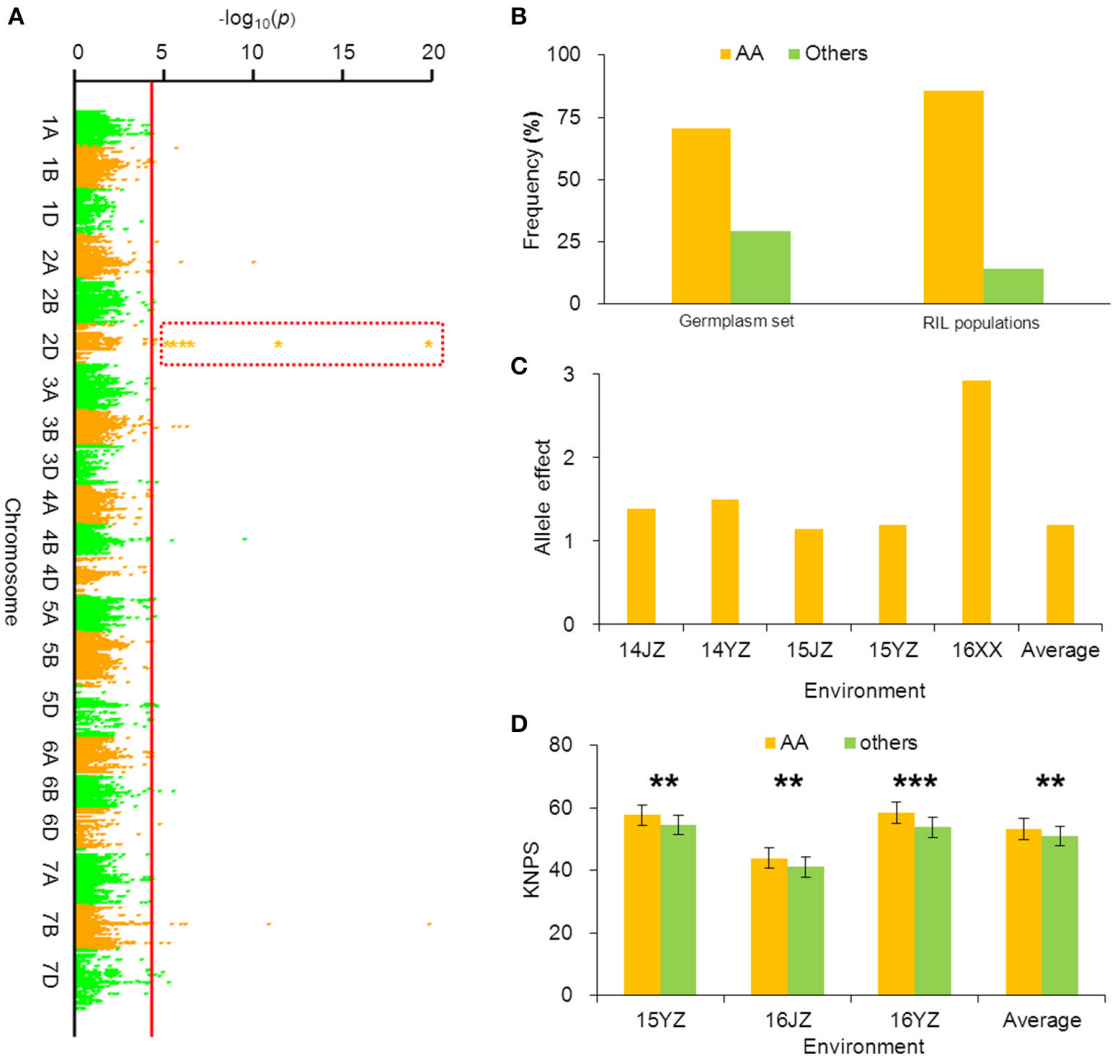
Favorable allele *BobWhite_c10539_201-2D*_*AA*_ associated with KNPS and analysis of its effect. **(A)** Markers associated with KNPS were identified by a mixed linear model in the germplasm set. The orange dots in the red frame show the association signals across all environments (*P* < 4.99 × 10^−5^). **(B)** Allelic frequencies (*AA* vs. *aa*) in the germplasm set and RIL population. Orange histogram represents the favorable allele. **(C)** Genetic effect of *BobWhite_c10539_201-2D*_*AA*_ in the germplasm set grown in different environments. **(D)** Genetic effects of *BobWhite_c10539_201-2D*_*AA*_ in the RIL population grown in different environments. ^**^P = 0.01; ^***^P = 0.001.

*BobWhite_c10539_201* was associated with KNPS in all environments in the cultivar population (Figure 3A, Table 2), and the favorable allele was AA. The frequency of this allele was 70.45% in the cultivar population and was obviously skewed in a positive direction, indicating strong selection during modern breeding (Figure 3B). The KNPSs in the cultivar population in each environment (14JZ, 14YZ, 15JZ, 15YZ, 16XX, BLUP) increased by 1.39, 1.50, 1.15, 1.19, 2.92, and 1.20, respectively (Figure 3C). Moreover, the average KNPS of the lines carrying the AA allele in all environments for the RIL population were significantly higher than that for lines carrying other alleles (*P* < 0.01; Figure 3D).

## Discussion

### Utilization of the 90K wheat iSelect SNP chip

SNP markers are the richest and ultimate point mutations in genomes, and are representative of ancient and stable variation. Genotyping using SNPs can be standardized, and the differences can be as simple as a single base pair. Moreover, SNP chips are highly-integrated, and can be combined with analysis software and relevant breeding information (Wang et al., 2014; Maccaferri et al., 2015). Thus, SNP markers can be used in a molecular breeding platform. Currently, applications of SNP chips in studies on QTLs in wheat are still at a preliminary stage. For instance, Thompson et al. (2015) used 9 K SNP chips to scan a wheat RIL population derived from the cross Louise/IWA8608077, and located QTLs associated with resistance to root-lesion nematode and root architectural traits using an established genetic map. Wang et al. (2014) used 90K high-density gene chips to scan eight DH wheat populations and built a SNP-integrated genetic map with an average distance of 0.09 cM. Jin et al. (2016) built a genetic map for a Gaocheng 8901/Zhoumai 16 RIL population consisting of 46,961 SNPs using 90 and 660K high-density gene chips. The total chromosome length was 4,121 cM with an average marker distance of 0.09 cM.

The 90K wheat iSelect SNP chip used in this study consisted of 81,587 SNP markers and scanned 34,039 polymorphic markers in the cultivar population, with a polymorphism frequency of 41.72%. Finally, 7,320 polymorphic markers were scanned in the RIL population, with a polymorphism frequency of 8.97%. Since the marker sequences of this SNP chip were known, sequence alignment can be used in evaluating marker effectiveness. Future work will enable construction of a high-density genetic linkage map for the RIL population.

### Integration of GWAS and bi-parental linkage analysis

Association analysis and traditional linkage mapping can be used in a complementary manner for gene identification and validation (Nordborg and Weigel, 2008). Using germplasm and RIL populations of soybean, Korir et al. (2013) detected five loci associated with aluminum resistance in both populations. The combination of the two methods improved the efficiency of screening for aluminum resistance candidate genes in soybean. Li et al. (2014) detected 22 seed weight and silique length-related QTLs in rape using a bi-parental population. Loci *uq.A09-1* and *uq.A09-3* were significantly associated in a germplasm population grown in multiple environments. Maccaferri et al. (2016) identified three major QTL clusters controlling root length and mass, including *RSA_QTL_cluster_5#, RSA_QTL_cluster_6#*, and *RSA_QTL_cluster_12#*, in two RIL populations and a germplsm set of durum wheat. The sequences surrounding these QTL will permit functional marker development and gene cloning. To validate association loci detected in a cultivar set we used the same SNP markers to scan the Yangmai 13/C615 RIL population. Among the 47 associated loci, 12 (25.53%) were significantly associated (*P* < 0.05) with KNPS in the RIL population grown in a different set of environments. In particular, *BS00022896_51-2A*_*TT*_, *BobWhite_c10539_201-2D*_*AA*_, *Excalibur_c73633_120-3B*_*GG*_, and *Kukri_c35508_426-7D*_*TT*_ were significantly associated with KNPS in all conditions (Table 4).

Among the 47 associated loci 35 (74.47%) were not validated in the RIL population. The main reason for this was the restricted bi-allelic polymorphism in a single segregating population. New strategies for combining linkage mapping and association analysis have been reported. For example, nested association mapping (NAM) is considered the most effective method to explain the genetic basis of quantitative traits for low-level LD species. NAM more effectively and economically scans at the genome-wide level, and helps to integrate molecular variation at the molecular level with that of complex phenotypic traits (Maurer et al., 2016; Saade et al., 2016).

In this study with the iSelect wheat 90K SNP chip we combined data from association mapping based on a germplasm set with linkage analysis of a RIL population to discover and validate QTLs for KNPS in wheat. Our findings theoretically permit cloning of candidate genes for KNPS. Moreover, the more important loci discovered here can be preferentially targeted in marker-assisted selection for high yield. Thus, the combination of association analysis and linkage mapping, development and application of powerful statistical models, and application of high-density SNP markers will promote research on the genetics of complex quantitative traits in crop species.

### Co-localization of QTLs/genes for yield-related traits

In this study, 12 of the 47 loci were validated to be correlated with KNPS in both populations. 9 of the 12 MTAs co-localizing with previous QTLs or loci were identified. They were located on chromosomes 2A (2), 2B (2), 2D, 3B, 4A, 7A, and 7B, respectively. The SNPs *GENE-1086_1111-2A, BS00022896_51-2A*, and *BobWhite_c10539_201-2D* on homologous group 2 were mapped to intervals of *QChl-A.caas-2AS* (*wsnp_Ex_c322_624793*-*Tdurum_contig10785_103*), *QChl-A.caas-2AL.2* (*Excalibur_c84687_162*-*BS00014251_51*), and *QChl-A.caas-2DS* (*BS00081578_51*-*tplb0021c10_951*) affecting SPAD value of chlorophyll content at anthesis, respectively (Gao et al., 2015). Besides, Gao et al. (2015) detected a QTL *QKNS.caas-2B.1* for KNPS on chromosome 2B in two environments in the bi-parental mapping population Zhou 8425B/Chinese Spring, and the authors assumed it to be the *Ppd-B1* locus. It is possible that *BS00014345_51* is tightly linked to the *Ppd-B1* gene (Beales et al., 2007). *Wsnp_JD_c12346_12606967* was mapped to *QGy.ubo-2B* in an interval of 97.4-150.0 cM, affecting grain yield in the RIL population (Milner et al., 2016). On chromosome 3B, SNP-marker *Excalibur_c73633_120* was significantly associated with KNPS in two environments (Table 2). This marker co-segregated with *BS00074688_ 51* that was related to days to heading by GWAS analysis in Pakistani historical wheat cultivars (Ain et al., 2015). Similarly, the SNPs *tplb0051b16_1324-4A, wsnp_BF474379A_Ta_2_2-7A*, and *BS00003630_51-7B* were also related to the corresponding QTLs, including *QSL.caas-4AS* (*Kukri_c46057_646*-*RAC875_rep_c77874_269*) coincided with spike length, *QMd.ubo-7A.3* (*IWB319*-*IWB23989*) with days to maturity and *QPh.ubo-7B* (*IWB10498*-*IWA7330*) with plant height, respectively (Gao et al., 2015). In addition, the SNP *Kukri_c35508_426* on chromosome 7D may close to the gene of *TaGS-D1* regulating grain weight and grain length, but more work will be needed to confirm (Zhang et al., 2014).

### Implications for molecular design and breeding based on KNPS

Breeding is a process of combining favorable alleles (Ge et al., 2011; Wang et al., 2012). So far, there is limited research on discovery of wheat yield QTL using the iSelect wheat 90K SNP chip. Gao et al. (2015) used an F_8_ RIL population developed from Zhou 8425B/Chinese Spring and detected 11 KNPS QTLs distributed in chromosomes 1B, 2A, 2B, 2D, 3A, 3B, 4A, 4B, 6B, and 7B. Three loci, *QKNS.caas-4AL* (*Kukri_rep_c106490_583-RAC875_c29282_566*), *QKNS.caas-3AL* (*RAC875_c61934_186-wsnp_Ex_c45877_51547406*), and *QKNS.caas-3B* (*RAC875_c10909_1180-BobWhite_c22016_155*) were detected in multiple environments. In the present study five associated loci *BS00022896_51* (2A), *BobWhite_c10539_201* (2D), *Excalibur_c73633_120* (3B), *BS00063906_51* (6B), and *GENE-4456_153* (7B) were significantly associated with KNPS in two or more environments (Table 2). The frequencies of *BS00022896_51-2A*_*TT*_*, BobWhite_c10539_201-2D*_*AA*_, *Excalibur_c73633_120-3B*_*GG*_, *BS00063906_51-6B*_*GG*_, and *GENE-4456_153-7B*_*TT*_ in the analyzed population were 64.82, 70.45, 92.82, 50.00, and 41.29%, respectively (Table 3). The skewed values imply that these alleles might have undergone selection during breeding.

## Author contributions

Conceived and designed the experiments: JG, PG, and SC. Performed the experiments: WS, YZ, and JC. Analyzed the data: JG and WS. Contributed reagents/materials/analysis tools: XY, JL, ZZ, XC, YX, DS, and XZ. Wrote the manuscript: JG, WS, and CH.

### Conflict of interest statement

The authors declare that the research was conducted in the absence of any commercial or financial relationships that could be construed as a potential conflict of interest.
